# Supplementary material for: New Insights into the Lactic Acid Resistance Determinants of Listeria monocytogenes Based on Transposon Sequencing and Transcriptome Sequencing Analyses
Source: Microbiol Spectr. 2022 Dec 21;11(1):e02750-22. doi: 10.1128/spectrum.02750-22 (PMC9927151; doi:10.1128/spectrum.02750-22)
Supplement: Supplemental file 1 — Table S1. Download spectrum.02750-22-s0001.pdf, PDF file, 0.05 MB [file spectrum.02750-22-s0001.pdf]

Supplementary material

Table S1. Primers used in this study

Primer	Sequence
pAT392_lox66_genta_F	TACCGTTCGTATAGCATACATTATACGAAGTTATGATAA ACCCAGCGAACCATTTGAGG
pAT392_lox71_genta_R	TACCGTTCGTATAATGTATGCTATACGAAGTTATTCAATC TTTATAAGTCCTTTTATAA
lmo2248_up_F	AAGCTGAATTCCTGCAGCCCTTGCCTTCTCTACAGCAC CAG
lmo2248_up_R	TGTATGCTATACGAACGGTACTTGAGGTAACGTTCTGA CAGCGTTTCC
lmo2248_down_F	GCATACATTATACGAACGGTAAGCTACACACCCTCGTC ATCGTACTTGC
lmo2248_down_R	CTAGAACTAGTGGATCCCCCCGGCGATGGAACCAATCA AG
Check_lmo2248_F	CGGTAACAGTGGATGCGTTT
Check_lmo2248_R	CGATTGGTCGTGGCTAAGTT
Comp_lmo2248_F	CTCGCATGCGAATTCCTGCAGCAAGTACGATGACGAG GGT
Comp_lmo2248_R	ACCACTAGTCCCGGGCTGCAGGAAACGCTGTCAGAAC GTT
